# Energy Metabolism and Intermittent Fasting: The Ramadan Perspective

**DOI:** 10.3390/nu11051192

**Published:** 2019-05-27

**Authors:** Nader Lessan, Tomader Ali

**Affiliations:** Imperial College London Diabetes Center (ICLDC), Abu Dhabi 48338, UAE; tfali@icldc.ae

**Keywords:** fast, intermittent, Ramadan, energy expenditure, weight

## Abstract

Intermittent fasting (IF) has been gaining popularity as a means of losing weight. The Ramadan fast (RF) is a form of IF practiced by millions of adult Muslims globally for a whole lunar month every year. It entails a major shift from normal eating patterns to exclusive nocturnal eating. RF is a state of intermittent liver glycogen depletion and repletion. The earlier (morning) part of the fasting day is marked by dominance of carbohydrate as the main fuel, but lipid becomes more important towards the afternoon and as the time for breaking the fast at sunset (iftar) gets closer. The practice of observing Ramadan fasting is accompanied by changes in sleeping and activity patterns, as well as circadian rhythms of hormones including cortisol, insulin, leptin, ghrelin, growth hormone, prolactin, sex hormones, and adiponectin. Few studies have investigated energy expenditure in the context of RF including resting metabolic rate (RMR) and total energy expenditure (TEE) and found no significant changes with RF. Changes in activity and sleeping patterns however do occur and are different from non-Ramadan days. Weight changes in the context of Ramadan fast are variable and typically modest with wise inter-individual variation. As well as its direct relevance to many religious observers, understanding intermittent fasting may have implications on weight loss strategies with even broader potential implications. This review examines current knowledge on different aspects of energy balance in RF, as a common model to learn from and also map out strategies for healthier outcomes in such settings.

## 1. Introduction

Fasting can be defined as the voluntary abstinence from or reduction of some or all food, drink, or both (absolute) for a period of time lasting typically between 12 h and 3 weeks i.e., in a short term, long term/prolonged or an intermittent pattern [1]. Fasting is a common practice in different religious disciplines, including Islam, Christianity, Judaism and Hinduism. In Islam, the practice entails abstinence from eating and drinking between dawn and sunset [2]. Fasting is distinct from starvation, which is a chronic and severe deficiency in caloric energy intake below the level needed to maintain life.

Health benefits of intermittent fasting have been demonstrated in both randomized controlled trials and observational studies [3,4]. Caloric restriction (CR) has also been shown to prevent several chronic degenerative and inflammatory diseases [5] and to prolong life in more primitive species including *Escherichia coli* and yeast [6]. In humans, the evidence on the positive effects of CR on longevity is indirect; for example the increased life expectancy in the Okinawan population, from the Kyushu Island in Japan, has been attributed at least in part to low calorie intake [7]. Mechanistically, the effect of CR on longevity has been attributed to fasting-induced modulation of neuroendocrine systems, hormetic stress responses, increased systemic production of neurotrophic factors, reduced mitochondrial oxidative stress, decreased pro-inflammatory cytokine production and insulin resistance, as well as decreased aging-associated signals and autophagy promotion [5,8,9].

Prolonged fasting has also been associated with positive effects on mood due to the alteration in physiology at a cellular level via increases in availability of central endogenous neurotransmitters, endogenous opioids and endocannabinoids [10]. Cancer studies demonstrated that fasting and fasting-mimicking diets (FMDs) positively promote differential effects in both normal and malignant cells via reduction in insulin-like growth factor (IGF-1), insulin and glucose with paralleled increases in ketone bodies [11]. In contrast, negative effects of fasting have been reported for instance on non-communicable diseases [8,11,12], on changes to sleep patterns, cognitive function, [13,14] and have also been associated with fluctuations in mood, weight and a plethora of other changes [15,16].

Fasting is a state of negative energy balance, and as such different fasting regimens have been used to achieve weight loss, as well as other health benefits. In the context of Muslim Ramadan-type fasting, changes in energy intake depend on social, cultural and individual factors and can range from a reduction to an increase in weight [17,18,19]. Whether this is accompanied by changes in energy expenditure is not well-known and merits further exploration for its possible implications in weight loss management strategies in general [20].

This review will be examining current knowledge about different aspects of energy balance in the context of the Ramadan fast as a commonly practiced model of intermittent fasting. In the broader context, potential positive implications include the use of for such strategies to help with weight maintenance, is not weight loss, and thus a multitude of other consequential positive health benefits. Relevant literature (Table 1 and Table 2) directly and indirectly related to the Ramadan fast, including short- and long-term fasting and also prolonged and intermittent type fasting will be explored. In the context of Ramadan, changes in energy dynamics (intake versus expenditure) have been extrapolated based on our previous quantitative studies, knowledge of physiology and alterations in energy utilization during feeding and non-feeding periods. The aim of this review is firstly, to discuss the various aspects influencing energy modulations during Ramadan fasting; secondly, to shed light on key knowledge gaps in our understanding of energy balance in relation to changes in both body composition and physiological adaptation in various models of fasting to include key periods such as the Ramadan fasting period and; lastly, to contribute to the focused directionality of future studies in key aspects that warrant further detailed investigations. 

## 2. Energy Expenditure (EE)

When body weight is in a relatively stable state, there is equilibrium between energy intake (EI) and energy expenditure (EE). High EI levels in combination with low EE results in a positive energy balance and storage of energy, primarily as body fat. Total (daily) Energy Expenditure (TEE) consists of Resting Metabolic Rate (RMR), Thermic Effects of Food (TEF) and Activity Energy Expenditure (AEE) [20]. Different components of EE have been reviewed elsewhere [21,22,23,24] and will only be discussed briefly here.

Resting Metabolic Rate (RMR) is the quantity of energy at rest needed to maintain body temperature, repair internal organs, support cardiac function, maintain ionic gradients across cells, and support respiration. In most people, this constitutes approximately two-thirds of total energy expenditure [25]. RMR is influenced by age, sex, body weight, pregnancy, and hormonal status. The highest rates of energy expenditure per unit of body weight occur during infancy and decline through childhood. In adult life, the decline continues at approximately 2% per decade because of a decline in lean body mass. Females have a lower energy expenditure per unit of weight than do males, probably because of the higher proportion of body fat and less lean body mass in women [26]. Thermic effect of food is the rise in energy expenditure that occurs with food intake [26]. This rise is in part due to the ‘obligatory’ energy cost of ingestion, digestion, and metabolic processing of nutrients, and in part due to a ‘facultative’ component arising from the sensory aspects of food and meal stimulation of the sympathetic nervous system. Different macronutrients have different thermic effects; protein causes a greater rise in EE than fat or carbohydrates. Although TEF is normally a small component of TEE (~10%) it is nonetheless an important component in energy imbalance states as it is influenced by meal size and composition, the nature of the previous diet, insulin resistance, physical activity, and ageing influence TEF [27].

### 2.1. Short-Term Fasting

Metabolically, fasting can be divided into three distinct key stages: Stage 1: a post-absorptive phase ~6–24 h after beginning fasting where the central nervous system (CNS) and many other issues preferentially use glucose produced from glycogen breakdown. Lipolysis and ketogenesis and gluconeogenesis increase, but the latter to a lower extent. Glycogenolysis decreases. Stage 2: the gluconeogenic phase occurs ~1–10 days after beginning fasting. Here, protein catabolism is used to feed glucose to the CNS while other tissues feed on ketones and fat. Lipolysis and ketogenesis increase and then plateau, gluconeogenesis on the other hand begins to decrease and no glycogenolysis occurs. Stage 3: is a protein conservation phase that occurs when fasting extends beyond 10 days. Protein catabolism is decreased to a minimum, fatty acids are used ubiquitously and ketones are utilized as fuel in the CNS. Lipolysis and ketogenesis plateaus while gluconeogenesis decreases and then plateaus but to a much lower extent when compared to ketogenesis [33,34].

### 2.2. Prolonged Fasting-Some Historic Examples

**Figure 1:**
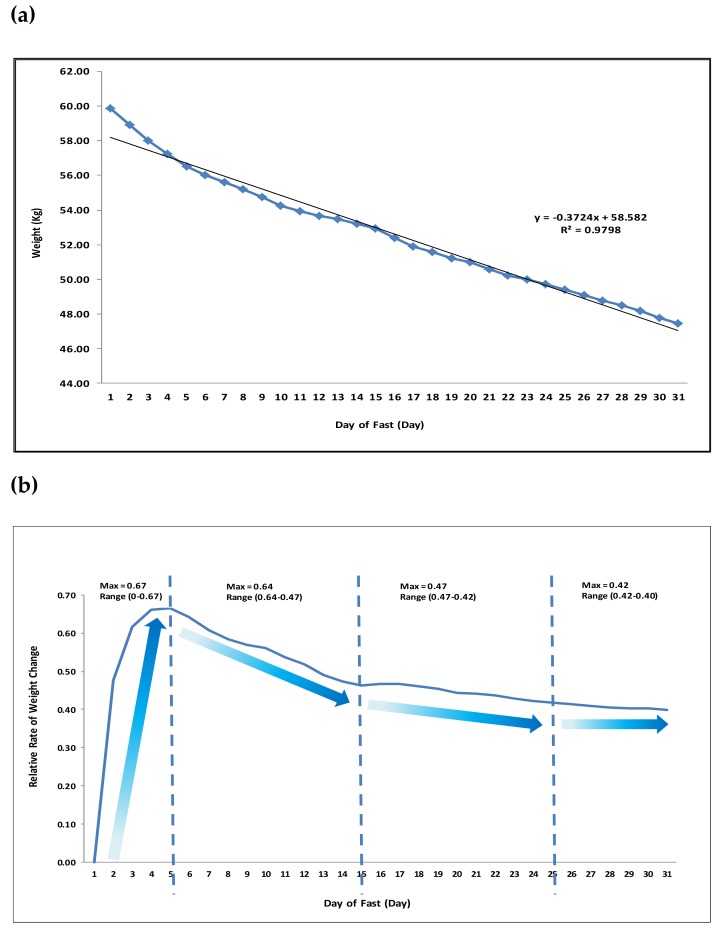
Time-dependent Changes in Weight during Prolonged Fasting (31 Days). Adapted from: Francis Gano Benedict: A study of Prolonged Fasting. (**a**), Daily Net Weight Loss: calculation of daily weight reduction in 31 days (D) of fasting. Initial weight was 59.86 kg at D1, final weight was 47.47 kg at D31, total weight loss −12.4kg. R^2^ = 9798 indicated a linear relationship between time and net weight loss. (**b**) Changes in Rate of Daily Weight Loss: relative to starting rate of weight loss, rate of weight loss per day indicates various changes whereby a steep rate of weight loss we observed in the first five days of fasting (D1–5; Maximum Rate 0.67), followed by a slower rate of weight loss in the following 10 days (D5–15; Maximum Rate 0.64), which decreased further in the next 10 days (D15–25; Maximum Rate 0.47) before reaching a plateau in the last five days of the fasting month (D25–30; Maximum Rate 0.42).

Early studies in 1915 by Francis Benedict looking into chemical and physiological alterations in a lean man fasting thirty-one days demonstrated significant declines in body weight (−12.4 kg with a rate of −0.84 kg/day at Day 1 declining to 0.32 kg/day by Day 31), (Figure 1). Levels of various biological markers such as body temperature and blood pressure were maintained [35,36]. 

In 1916, Spriggs reported various cases of fasting used as a method to treat diabetes whereby fasting was ‘continued in bed until the urine has been sugar-free for twenty-four hours, unless there is some definite contraindication, such as nausea, vomiting, insomnia, or faintness’ [37]. Early studies also indicated a progressive decrease in daily urinary nitrogen excretion suggestive of an increase in conservation of body protein [38] and that urine output gradually decreased throughout the fasting period [39]. 

In 2006, a study on prolonged absolute fast (44-days) on a healthy non-obese man shed light on changes in various metabolic parameters [40]. The TEE was not measured, but was estimated to be 1638–2155 kcal/day of which 13.0–17.1% was from protein oxidation. Total weight loss was 24.5 kg and body mass decreased by 25.5%; a quarter to a third was fat mass and the remainder to fat-free mass which was predominantly muscle and approximately 20% was total body protein. 

More recently, in 2015, Müller and colleagues investigated effects of caloric restriction (CR) and weight loss on 32 subjects aged between 20–37 years old in a controlled environment. Patterns of habitual food intake, resting energy expenditure and physical activity were assessed. The 10 week (week) dietary intervention period duration included 1 week of overfeeding (at +50% of daily energy requirements; 4059 ± 52 kcal/day) followed by 3 weeks of CR (at −50% of energy requirements; 1353 ± 154 kcal/day) and a subsequent 2 weeks of re-feeding (at +50% of energy requirements; 4059 ± 452 kcal/day). Protein intake was 97 ± 11 g/day (baseline); 146 ± 17 g/day (overfeeding), 49 ± 6 g/day (CR), and 146 ± 17 g/day (re-feeding), respectively. The study reports a +1.8 kg weight gain (overfeeding), −6.0 kg (CR), and +3.5 kg (re-feeding). CR reduced fat mass and fat-free mass from skeletal muscle (−5%), liver (−13%), and kidneys (−8%) by a total of 114 and 159 g/day, respectively. CR also led to reductions in resting energy expenditure (−266 kcal/d) and respiratory quotient (−15%). The study concluded that during early weight loss, adaptive thermogenesis is associated with a fall in insulin secretion and body fluid balance [41].

## 3. The Ramadan Fast: A Shift from Normal Eating Patterns

**Figure 2:**
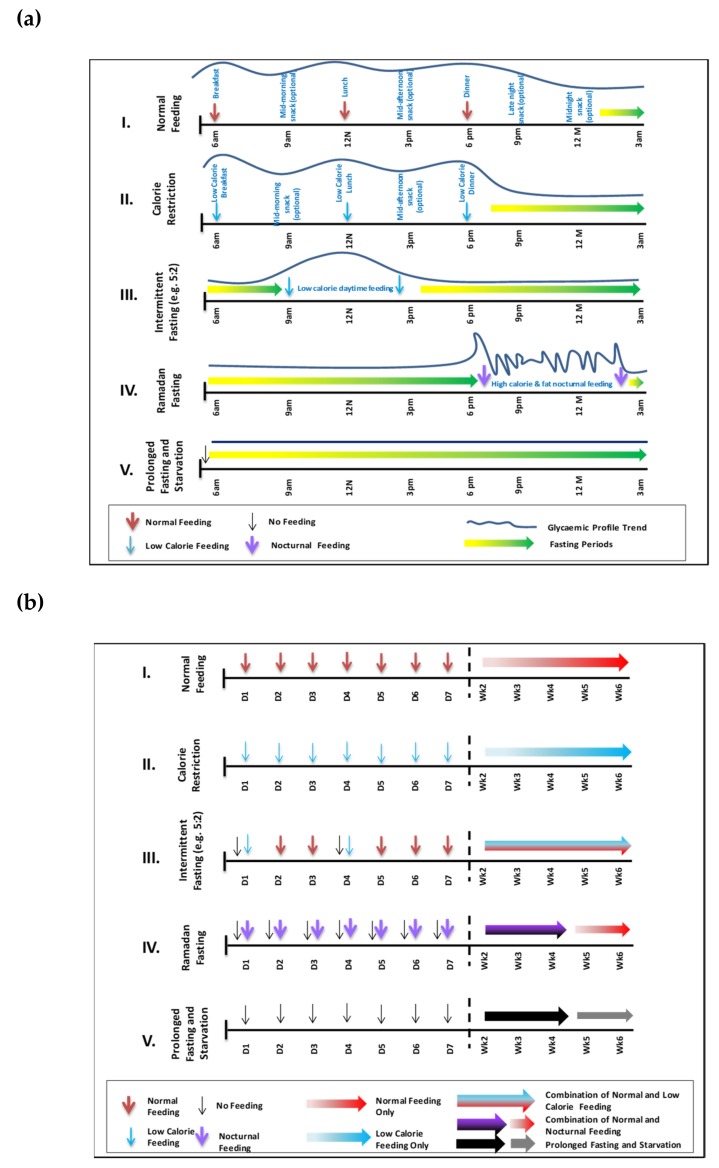
Changes in Feeding Patterns and Energy Intake during Various Fasting Periods. The five feeding and fasting patterns are: (I) normal feeding, (II), calorie restriction, (III) intermittent fasting (e.g., 5:2), (IV) Ramadan fast and (V) prolonged fasting and starvation. (**a**) Hourly Differences in Feeding Patterns between Various Fasting Models: hourly timings of feeding and energy intake (meals) are indicated per day in relation to fasting periods (arrows) and reflected in glycaemic control (traces). (**b**), Daily and Weekly Differences in Feeding Patterns Between Various Fasting Models: daily and weekly feeding patterns are mapped against calorie intake which can be regular such as in in normal feeding (I), indicated by single colour arrows or a combination of low, normal or high calorie intake as in intermittent fasting (III), indicated by mixed colour arrows. Ramadan fast (IV) is unique as it combined low and high calorie intake as indicated by the two single colour arrows. The first week is broken down into seven individual days. Weekly indications follow thereafter.

A typical eating pattern in most cultures includes three main meals, often accompanied by snacks in between (Figure 2). Alterations in this ‘normal’ pattern can have important implications to energy balance. Some of the more common fasting regimens include intermittent fasting (IF), periodic fasting (PF) and time restricted fasting (TRF) [8]. 

Ramadan fasting and Ramadan-type fasting are somewhat different from other forms of fasting mentioned above. Ramadan, the ninth month in the Islamic Calendar, requires Muslims to fast daily from dawn to dusk and the criteria are clearly defined in the Holy Quran [2]. No food or drink is allowed after suhoor until iftar. The fast is traditionally broken with something sweet such as dates. This is followed by the main meal which tends to be heavy and carbohydrate-rich. Between iftar and suhoor, food can be taken without any restriction. Ramadan is a lunar month and as such lasts 29-30 days. The fast is a religious obligation for all adult Muslims. Exempt groups include the sick and also women during their menstrual period. Many people who are religiously exempt opt to fast, often for social and cultural reasons.

In addition to Ramadan fasting, many Muslims practice the same dawn-to-sunset type of fast on other days of the year and this may include Mondays and Thursdays. Fasting some days may have some physiological differences from fasting an entire month as some physiological adaptations which may happen later during Ramadan may not occur in the short term. 

## 4. The Ramadan Diet

**Figure 3:**
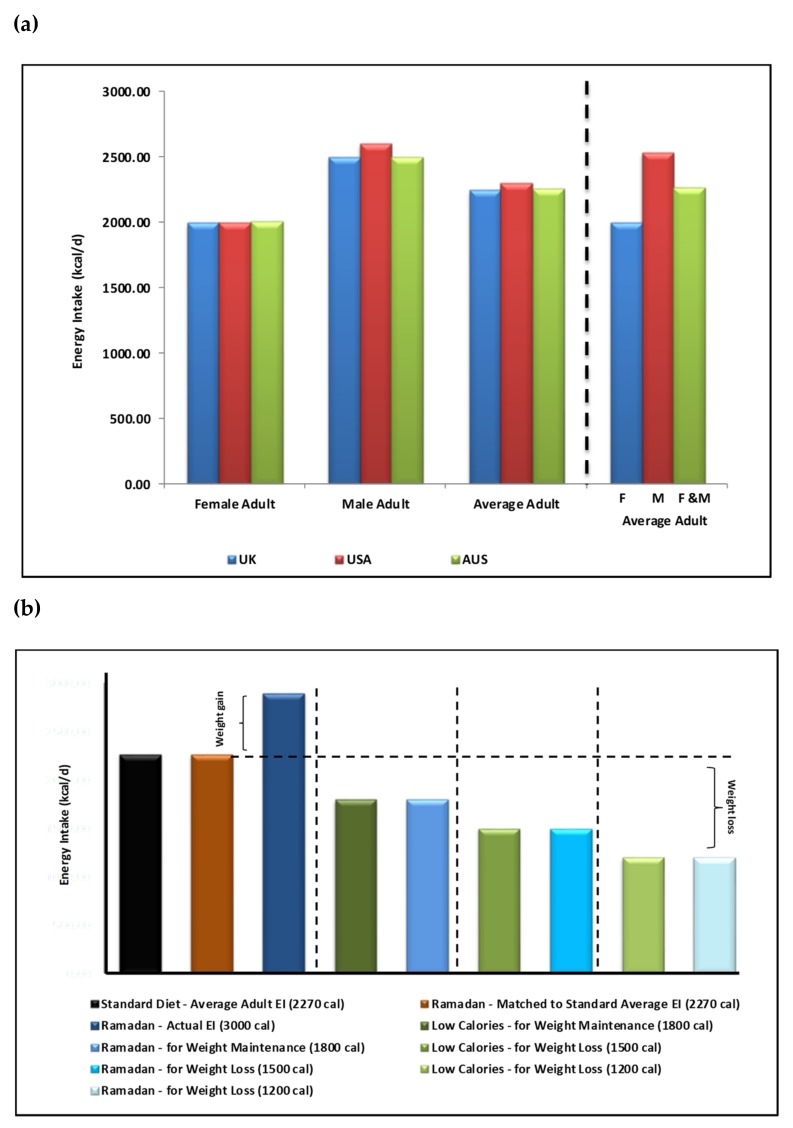
Energy intake (EI) recommendations and resultant weight changes in Ramadan and non-Ramadan periods. Energy intake recommended guidelines for female and male adults. (**a**) indicates values for the (1) UK 2250 kcal/day (female 2000 and male 2500 kcal/day), (2) the USA 2300 kcal/day (female 2000 and male 2600 kcal/day) and (3) for Australia 2225 kcal/day (female 2010 and male 2600 kcal/day). Collectively, an average adult consumes ~2270 kcal/day (female 2003 and male 2533 kcal/day). (**b**), Energy intake recommendations during Ramadan in comparison to standard and low calorie diets. in order of left to right: based on the calculated average of 2270 kcal/day as a standard adult EI (Figure 3A), a healthy Ramadan diet matched calorie intake is achievable. In reality, a higher EI is experienced in Ramadan (~3000 calories). However, weight maintenance (at 1800 kcals/day) is achievable during Ramadan as suggested by Diabetes and Ramadan (DaR) Alliance Ramadan Nutrition Plans (RNP) recommendations. This holds true for weight loss at the 1500 and 1200 kcals/day calorie EI for both non-Ramadan and Ramadan periods.

Management of a healthy balanced diet is necessary not only for the maintenance of a healthy weight, but for the maintenance of the overall nutritional health of individuals too. Energy intake plays a central role [42]. Nonetheless, multiple factors influencing energy intake such as cultural and lifestyle differences, make it difficult to maintain healthy balanced diet long-term. During non-fasting periods, recent statistics indicate that average daily adult energy intake is: (1) 2250 kcal/day (female 2000 and male 2500 kcal/day) in the UK [43], (2) 2300 kcal/day (female 2000 and male 2600 kcal/day) in the USA [44] and 2255 kcal/day (female 2010 and male 2600 kcal/day) in Australia [45]. Collectively, an average adult consumes ~2268 kcal/day (female 2003 and male 2533 kcal/day) (Figure 3A) with an additional margin for genetic (e.g., predisposition to overweight/obesity) and environmental influences (e.g., daily activity and feeding habits). 

Ramadan nutrition planning (RNP) is encouraged as per DaR guidelines, which take into consideration variations in cultural food choice and calorie consumption (range of 1200 kcal/day for weight reduction for females to maximum of 2000 kcal/day weight maintenance for males) [46]. Due to the inevitable changes in feeding patterns and associated physiological shifts in circadian rhythms, hormone levels fluctuations and overall daily lifestyle, Ramadan meal planning becomes an essential component for healthy Ramadan fasting. This is of particular importance for patients with chronic conditions, such as diabetes. A ‘Ramadan Plate’ is recommended to contain a balanced selection of carbohydrates (40–50% of total daily calorie intake (TDCI) of low glycaemic index and high-fibre containing foods), protein (20–30% of TDCI of non-red meat sources and legumes) and reduced fat intake (35% of TDCI of mostly mono- and poly-saturated fatty acids). Suhoor, the pre-dawn meal, is recommended to constitute 30–40% energy intake for the day, iftar 40–50% and snacks 10–20% as necessary.

In theory, in terms of energy intake, skipping one main meal in a 24-h period should be associated with a major reduction in food content and energy intake. This is the principle in the intermittent 5:2 fasting diet where fasting can be up to 18 h (Figure 2(AIII,BIII)). Therefore, during Ramadan, in addition to eating healthily, this reduction in energy intake could lead to weight loss but in practice this does not occur in most cultures (Figure 3B). Many studies indicate a great variability in Ramadan diets [30,47] in different cultures, age groups, geographical locations and duration of fasting hours as well as the impact of physiological and pathological conditions (e.g., diabetes) and associated with modest reduction of energy intake in most but not all groups studied. 

El Ati and colleagues investigated a group of 16 healthy female volunteers fasting during Ramadan and reported 84% of total daily energy intake was taken at the evening meal, and the remaining 16% was taken between 8 p.m and midnight. This is in contrast to periods before Ramadan where breakfast, lunch and dinner constituted 9.4, 41.6 and 21.8% of total daily energy intake. Although the findings of this small study cannot be generalized to the larger population of fasting Muslims, the observation of a disproportionately large meal at iftar time is a common finding [31,48]; often reflected in feeding patterns (Figure 2) and in glycaemic profiles.

## 5. Weight and Body Composition Changes During Ramadan Fasting

There seems to be much inter-individual variability in weight trends with Ramadan fasting and as with other modalities of weight change; one would expect these to be determined by individual, cultural and social factors as well as genetic, epigenetic and other factors such as gut microbiome. Several small studies (with participants between 16–81 years old in most) have examined the effect(s) of Ramadan on body weight and reported a modest weight loss of 1–2 kg by the end of Ramadan, with some other studies reporting weight gain [18,19]. A meta-analysis of the older studies (by Kul et al., 2014) showed a small weight loss of around 0.7 kg in fasting men, but no significant change in fasting women [18]. The largest study of 202 participants (Hajek et al., 2012) recruited at mosques in East London showed a net weight loss of around 0.8 kg by the end of Ramadan [49]. As in some other studies that had post-Ramadan weight recorded, this study showed that all the lost weight was regained 4–5 weeks after Ramadan [18]. In terms of satiety and hunger, the levels remained the same for males during Ramadan while for females more hunger was experienced earlier in the month and then decreased as the Ramadan month progressed [30,50].

In a more recent excellent meta-analysis, Fernando and colleagues showed that the mean weight loss with Ramadan fasting was 1.34 kg and that most of the weight was regained a few weeks post-Ramadan [51]. It has also been shown that weight loss is greater among Asian populations compared with Africans and Europeans [19] and that there does not appear to be any gender difference in the absolute magnitude of weight loss with Ramadan fasting.

## 6. Energy Expenditure During Ramadan Fasting

### 6.1. Resting Metabolic Rate (RMR) During Ramadan Fasting

RMR is known to decrease with prolonged fasting and this may be a counter-regulatory way to decrease energy loss (Benedict, 1915; Forbes, 1987; Garrow, 1978; Woo et al., 1985) [26]. Studies into RMR changes in Ramadan are however few in number. A study by El Ati and colleagues (1995) is the earliest reported [28]. Different aspects of EE in the context of the Ramadan fast in 16 female participants were explored. RMR at four different time points around Ramadan (before Ramadan, the first week of Ramadan, the last week of Ramadan, and the month after Ramadan) were investigated along with daily EE trends. The study reported a reduction in RMR during Ramadan and metabolic rate patterns were found to be different between Ramadan and non-Ramadan days; lower during the fasting day versus night, with a rise around iftar, but no significant change overall. Total daily energy intake, body weight, fat mass and fat free mass remained unchanged. Similarly, a study by Bahammam and colleagues found a reduction in EE and metabolic equivalents (METs) measured by accelerometry during Ramadan fasting [14]. In our own study of 45 male and female subjects we found no overall difference in RMR between Ramadan and non-Ramadan periods (mean ± SD: 1365 ± 230 compared with 1363 ± 274 kcal/day for Ramadan and post-Ramadan respectively, *p* = 0.713, *n* = 29). However, multiple linear regressions and controlling for the effects of age, sex, and body weight, RMR was higher in the first week of Ramadan and showed a significant downward trend in subsequent weeks [24] potentially due to metabolic adaptation medicated both centrally and locally (e.g., via gut hormones). Thorough investigations, particularly in the context of Ramadan, need to be conducted to more accurately and precisely assess the contribution of these individual factors in fasting-related energy regulation. 

### 6.2. Activity Energy Expenditure (AEE) During Ramadan Fasting

**Figure 4:**
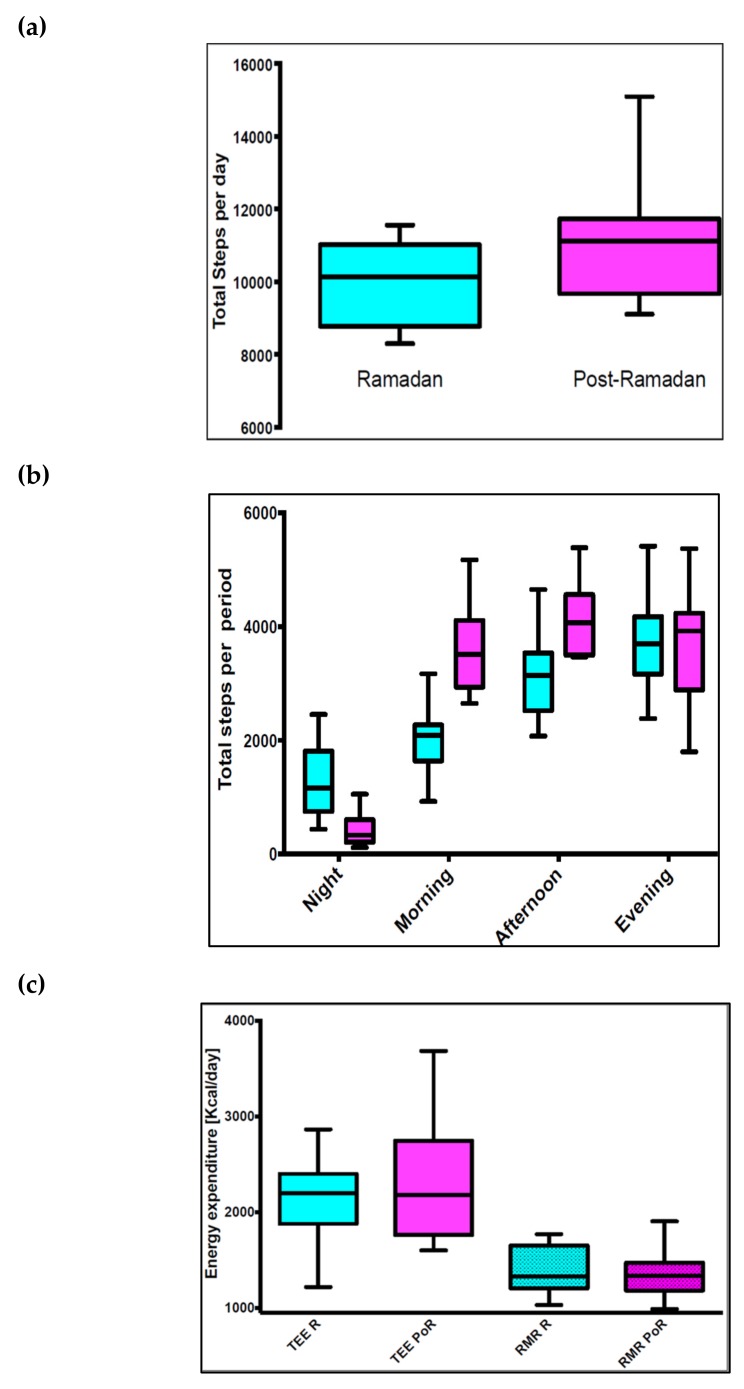
Energy expenditure and physical activity pre-, during and post-Ramadan. (**a**) Box plot of daily total number of steps during and post-Ramadan. The effect of Ramadan fasting on activity in 11 participants. (**b**) Box plot of total number of steps at different periods within one day (per night, morning, afternoon, and evening) during and post-Ramadan in 11 participants. Comparisons made with the Wilcoxon signed-rank test. Total mean ± SD number of steps per day (9950 ± 1152 compared with 11,353 ± 2053, *p* = 0.001), activity in the morning (1974 ± 583 compared with 3606 ± 715, *p* = 0.001) and afternoon (3193 ± 783 compared with 4164 ± 670, *p* = 0.002) were significantly lower during Ramadan compared with post-Ramadan. Nocturnal activity was higher during Ramadan (1261 ± 629 compared with 416 ± 279, *p* = 0.001). No significant difference in evening activity levels between during and post-Ramadan periods was observed. (**c**) TEE and RMR during and post-Ramadan: the correlation between TEE and weight during and post-Ramadan in 10 participants. No significant difference between Ramadan and post-Ramadan regression lines (ANCOVA; *t* = 0.35, *p* = 0.727); the main factor influencing TEE was body weight (*t* = 2.72, *p* = 0.015).

As well as changes in meal times and content, Ramadan period is associated with major changes in activity patterns throughout the fasting day. Much of the daily activity and AEE tends to occur nocturnally after iftar [52] with inter-individual variability reported in various other studies [32,52,53]. A recent study by our group (Lessan et al., 2018) investigated daily activity patterns using accelerometers an overall reduction in daily activity energy expenditure [24]. Differences in daily activity patterns between Ramadan and non-Ramadan periods were observed (Figure 4). Activity in the morning (1974 ± 583 compared with 3606 ± 715, *p* = 0.001) and afternoon (3193 ± 783 compared with 4164 ± 670, *p* = 0.002) were significantly lower during Ramadan compared with post-Ramadan. Nocturnal activity was higher during Ramadan (1261 ± 629 compared with 416 ± 279, *p* = 0.001). No significant difference in evening activity levels between during and post-Ramadan periods was seen however the study found a reduction in activity during fasting hours and a rise after iftar. Furthermore, major change in sleeping patterns and times was reported. 

### 6.3. Thermic Effect Of Food (TEF) During Ramadan Fasting

There have been no studies specifically investigating TEF in the context of Ramadan fasting and it is difficult to speculate how TEF would change with the Ramadan fast. However, there are a number of considerations in speculating what changes to TEF might be expected with the Ramadan fast. Firstly, TEF is related to serum insulin and insulin resistance. Insulin resistance and plasma insulin level are known to be higher during the Ramadan fast, especially in the evening and around iftar period. This may lead to a reduction in TEF. Secondly, dietary fat has a lower thermic effect than protein. Several studies of diet during Ramadan have indeed, reported a higher fat content; this can also cause a reduction in TEF during Ramadan. Finally, a major meal is skipped during Ramadan, and although this can in part be compensated by over-snacking at nights, a net reduction in TEF may be expected. Well-conducted studies of TEF during Ramadan can provide a better insight into energy dynamics during Ramadan and help with weight management around the Ramadan period.

### 6.4. Fuel Utilization During Ramadan Fasting

Few studies have investigated fuel utilization in the context of the Ramadan fast. Using indirect calorimetry, El Ati has shown that during Ramadan fat oxidation increases through the fasting day. Carbohydrate oxidation decreases gradually from morning to iftar time. The differences in fuel oxidation at different time points in Ramadan and non-Ramadan days were significant [51]. AlSubheen and colleagues have also shown that carbohydrate oxidation drops and lipid oxidation gradually increases through the Ramadan fasting day [29].

### 6.5. Total Energy Expenditure (TEE) During Ramadan Fasting

El Ati and colleagues reported measurements of energy expenditure by indirect calorimetry at several time points through Ramadan and non-Ramadan days reportedly in a metabolic chamber. No total energy expenditure values were however reported. Substrate oxidation and biochemical assays were also carried out over the four-day test period between 8 a.m. and 11 p.m. at three hourly intervals. The study reports that resting energy expenditure measured at 8 a.m. remained unchanged during and after Ramadan, compared to pre- Ramadan durations. However, the EE throughout the circadian cycle was dramatically affected during Ramadan fasting periods whereby, and unlike the nightly energy expenditure values, a significant decrease in energy expenditure was observed from 11 a.m. to 5 p.m. hours during Ramadan fasting periods [13]. 

Our study in healthy non-obese volunteers investigated changes in RMR and TEE in free-living conditions. The study of TEE utilizing doubly-labelled water and accelerometer aided techniques by our group reported no differences in TEE between Ramadan and Post-Ramadan periods (mean ± SD: 2224 ± 434 compared with 2121 ± 719 kcal/day for Ramadan and Post-Ramadan, *p* = 0.7695, *n* = 10) (Figure 4). TEE did not differ significantly between Ramadan and Post-Ramadan [24]. The insulin resistance observed [24] was a result of the compounding factors of reduction in circulating leptin, a gradual shift from carbohydrate to lipid as dominant fuel as the fasting day progresses and the variable weight change determined by individual, social and cultural factors, rather than physiological changes.

## 7. Discussion and Concluding Remarks

Calorie restriction and different forms of fasting have been shown to have major physiological effects; from health benefits to longevity [6,54]. Ramadan fasting has also been shown to have beneficial effects including positive changes in body composition with reported reduction in body fat as well as weight loss which is a common although not universal consequence [50]. Similar to calorie-restricting diets targeting calorie reduction at ~500–800 kcal/day [55], skipping a meal during fasting, such as in the context of Ramadan, can theoretically lead to weight loss. However, dietary changes during Ramadan vary and often include an increase in carbohydrate intake [56,57]. 

Weight loss strategies including many dietary interventions are often unsuccessful in the medium and the long term. One explanation for this is the phenomenon of adaptive thermogenesis. This occurs by promoting optimization of energy reserves while preserving protein pools via reduction in basal metabolism, decrease in secretion of anabolic factors (e.g., insulin) and increase in catabolic hormones (e.g., adrenaline and glucagon) [3]. Along with protein loss, weight loss also occurs; initially at a higher rate (~1kg/day) which then decreases (~0.7 kg/day by 24 h, 0.5 kg/day by day 6 and 0.3 kg/day from day 21 onwards) [33]. Importantly, the few small studies of energy expenditure in the context of Ramadan fast have found no evidence of a metabolic adaptation [24]. This finding needs to be investigated in larger studies and if confirmed, may have important implications on Ramadan and IF as potential weight loss strategies. Admittedly, overcompensation with an increase in energy intake at the evening meal is common practice in observers of the Ramadan fast [31]. Although the increased appetite at the end of the fasting day [49] is the main drive for this phenomenon, this is in many ways voluntary. With appropriate education and a shift in food choices it may be possible to limit this increase in intake of energy dense food and make the prospect of weight loss with the Ramadan fast more realistic.

Aside from weight changes, Ramadan fasting induces a plethora of physiological and metabolic alterations. The impact of Ramadan on sleep alone includes decreased total sleep time, delayed sleep, decreased sleep period time (decreased REM sleep duration, decreased proportion of REM sleep) and increased proportion of non-REM sleep [13]; also reported with high inter-individual variation. 

An important issue on interpretation of Ramadan studies is the potential hypohydration that would be expected towards the end of the Ramadan fasting day. A study investigating the effects of prolonged fasting and fluid deprivation reported a loss of body weight of around 1.5 kg in individuals fasting between 10 pm and 4 pm the next day; the weight loss was presumed to be due to loss of body water [39]. Fluid homeostasis during Ramadan fast has been investigated in several studies and has been reviewed elsewhere [58]. Water turnover has been shown to increase during Ramadan fast with concomitant increases in indicators of body hydration including haematocrit, serum urea and creatinine and urine osmolality. However, total body water appears to be conserved and aside from potentially contributing to weight loss that might be observed in Ramadan, no detrimental effects on health have been directly attributed to negative water balance and hypohydration at the levels experienced during Ramadan [58]. Furthermore, hypohydration has been shown to have no significant effect on RMR and blood glucose in healthy subjects [59]. 

Studies of Ramadan fasting in general need to be interpreted carefully and with consideration for certain factors such as the timing of previous meal, methodological differences and also hydration status. An important and relevant factor in studies of Ramadan fasting is the duration of the fast, and hence geographical location; the impact tends to be most marked in countries at higher altitudes and with more daylight hours [60]. Fasting hours also include the seasonal changes whereby fasting Ramadan during winter months for instance would have physiologically different effects when compared to fasting Ramadan during summer months. Although the literature specifically pertaining to energy expenditure changes during Ramadan is steadily mounting, it is currently small in number. Therefore, future studies need to address these variables to tackle the inter-variability issues that continually arises in the current literature. 

In conclusion, although the metabolic consequences of Ramadan fast are complex, there is potential for using this month as a weight reduction model provided the fasting is carried out mindfully; balancing food type, quantity and levels of physical activity. Pre-Ramadan planning (nutrition plans, medication and health checks) is necessary; more so for individuals with chronic conditions such as diabetes who need specialist advice should Ramadan fast be deemed suitable in the first place. The long-term effects are thus of interest and studies are necessary for elucidation.

## Figures and Tables

**Table 1:** Energy Expenditure in Ramadan.

Ref.	Year	Study Cohort	Gender & Age (Years)	Reported Observations
[14]	2010	Healthy adults 1-week pre-Ramadan baseline (BL) as well as first and second week of Ramadan (R1) and (R2); *n* = 7).	Males; 21 ± 3	SenseWear Pro Armband measurements indicated EE and METs significantly lower during Ramadan and a shift in circadian patterns (of body temperature, a delay in bedtime and an increase in total sleep time and nap time) during Ramadan. No significant difference in the number of meals.
[24]	2018	Healthy adults during Ramadan and non-Ramadan periods. RMR (*n* = 29, 16 female) Activity (total steps per day) (*n* = 11, 5 female); TEE (*n* = 10, 5 female).	Female and male; 33 ± 9	Indirect calorimetry; (a) activity during and post- Ramadan; no significant difference, (b) activity pattern: morning & afternoon significantly lower during Ramadan. Nocturnal activity was higher during Ramadan, (c) TEE & RMR during and post-Ramadan: no significant difference; main factor influencing TEE was body weight.
[28]	1995	Healthy adults, 2 days pre-Ramadan (T1); the 2nd day (T2), and the 28th day (T3) of fasting; & 1 month after, (*n* = 16).	Female; 25–39	Indirect calorimetry; calculations from metabolic chamber; REE unchanged during and post-Ramadan, compared with pre- Ramadan. EE throughout the circadian cycle was dramatically affected during fasting with a significant decrease observed from 11am to 5pm during Ramadan. Nightly EE values did not change significantly.
[29]	2017	Healthy fasting (FAST, *n* = 9) and non-fasting (CNT, *n* = 8) adults pre and post-Ramadan. FAST group additionally assessed at days 10, 20 & 30 of Ramadan (am) & (pm).	Male; FAST: 32 ± 8 and CNT: 35 ± 9	Indirect calorimetry; significant group × time interaction, reduced body mass and adiposity in FAST, without changing lean mass; for CNT subjects, remained unchanged. Ramadan fasting induces diurnal metabolic adjustments (morning v. evening) with no carryover effect observed throughout Ramadan fasting despite the extended daily fasting period and changes in body composition.

**Table 2:** Energy intake and weight changes during Ramadan.

Ref	Year	Study Cohort	Gender & Age (Years)	Reported Observations
[18]	2014	Healthy fasting adults with normal body weight; *n* = 1476; 553 female and 923 male)	Female and male; ≥18	In the female subgroup, body weight (SMD = −0.04, 95% CI = −0.20, 0.12) remained unchanged, while in males, Ramadan fasting resulted in weight loss (SMD = −0.24, 95% CI = −0.36, −0.12, *p* = 0.001).
[28]	1995	Healthy fasting adults, two days pre-Ramadan (T1); second (T2) and 28th day (T3) of Ramadan; and 1 month post-Ramadan (T4); *n* = 16	Female; 25–39	Total daily energy intake, body weight, fat mass and fat free mass remain unchanged. REE pattern change; lower during the fasting day versus night but no significant change overall.
[29]	2017	Healthy fasting (FAST, *n* = 9) and non-fasting (CNT, *n* = 8) adults pre and post-Ramadan. FAST group additionally assessed at days 10, 20 & 30 of Ramadan both (am) and (pm).	Male; FAST: 32 ± 8, CNT: 35 ± 9	Significant group × time interaction revealed reduced body mass and adiposity in FAST, without changing lean mass, whereas CNT subjects remained unchanged. Although RF induces diurnal metabolic adjustments (morning v. evening), no carryover effect was observed throughout Ramadan fasting despite the extended daily fasting period and changes in body composition.
[30]	2009	Healthy fasting adults, *n* = 46; 24 female and 22 male.	Female and male; 24 ± 3	Total energy intake was higher during Ramadan (13 and 11 MJ/day) than before and after Ramadan (11 and 9 MJ/day) in men and women, respectively.
[31]	2011	173 families fasting Ramadan	Female and male; age unspecified	59.5% reported weight gain post-Ramadan; 40% attributed food types, 31.2% attributed to relative lack of physical exercise and 14.5% referred that to increase in food consumption. 65.2% of those with increased expenditure reported weight gain.
[32]	2007	Healthy fasting adults at one-week pre-Ramadan (T1), first week (T2), end of second week (T3), and end of last week (T4) of Ramadan; *n* = 57	Female; 22 ± 4	Body weight and BMI decreased significantly during Ramadan fasting. The mean physical activity level was not significantly different. The overall activity patterns remained similar; 1.54 pre-and 1.51 during Ramadan

## References

[1] Patterson R.E., Laughlin G.A., LaCroix A.Z., Hartman S.J., Natarajan L., Senger C.M., Martínez M.E., Villaseñor A., Sears D.D., Marinac C.R. (2015). Intermittent Fasting and Human Metabolic Health. J. Acad. Nutr. Diet..

[2] Ali M.M. (2011). The Holy Quran.

[3] Horne B.D., Muhlestein J.B., Anderson J.L. (2015). Health effects of intermittent fasting: Hormesis or harm? A systematic review. Am. J. Clin. Nutr..

[4] Jane L., Atkinson G., Jaime V., Hamilton S., Waller G., Harrison S. (2015). Intermittent fasting interventions for the treatment of overweight and obesity in adults aged 18 years and over: A systematic review protocol. JBI Database Syst. Rev. Implement Rep..

[5] Michalsen A., Li C. (2013). Fasting therapy for treating and preventing disease—Current state of evidence. Forsch. Komplementmed..

[6] Omodei D., Fontana L. (2011). Calorie restriction and prevention of age-associated chronic disease. FEBS Lett..

[7] Willcox B.J., Willcox D.C. (2014). Caloric restriction, caloric restriction mimetics, and healthy aging in Okinawa: Controversies and clinical implications. Curr. Opin. Clin. Nutr. Metab. Care.

[8] Mattson M.P., Longo V.D., Harvie M. (2017). Impact of intermittent fasting on health and disease processes. Ageing Res. Rev..

[9] Desgorces F.D., Breuillard C., Police C., Neveux N., Cottart C.H., Blanc M.C., Toussaint J.F., Noirez P. (2016). Short-term Effects of Diet and Activity Changes on Inflammation and Insulin Resistance. Int. J. Sports Med..

[10] Michalsen A. (2010). Prolonged fasting as a method of mood enhancement in chronic pain syndromes: A review of clinical evidence and mechanisms. Curr. Pain Headache Rep..

[11] Nematy M., Alinezhad-Namaghi M., Rashed M.M., Mozhdehifard M., Sajjadi S.S., Akhlaghi S., Sabery M., Mohajeri S.A., Shalaey N., Moohebati M. (2012). Effects of Ramadan fasting on cardiovascular risk factors: A prospective observational study. Nutr. J..

[12] Sahin S.B., Ayaz T., Ozyurt N., Ilkkilic K., Kirvar A., Sezgin H. (2013). The impact of fasting during Ramadan on the glycemic control of patients with type 2 diabetes mellitus. Exp. Clin. Endocrinol. Diabetes.

[13] Roky R., Chapotot F., Hakkou F., Benchekroun M.T., Buguet A. (2001). Sleep during Ramadan intermittent fasting. J. Sleep Res..

[14] BaHammam A., Alrajeh M., Albabtain M., Bahammam S., Sharif M. (2010). Circadian pattern of sleep, energy expenditure, and body temperature of young healthy men during the intermittent fasting of Ramadan. Appetite.

[15] Parnell J.A., Reimer R.A. (2009). Weight loss during oligofructose supplementation is associated with decreased ghrelin and increased peptide YY in overweight and obese adults. Am. J. Clin. Nutr..

[16] Patel S.R., Hu F.B. (2008). Short sleep duration and weight gain: A systematic review. Obesity.

[17] Haouari M., Haouari-Oukerro F., Sfaxi A., Rayana M.B., Kaabachi N., Mbazaa A. (2008). How Ramadan fasting affects caloric consumption, body weight, and circadian evolution of cortisol serum levels in young, healthy male volunteers. Horm. Metab. Res..

[18] Kul S., Savaş E., Öztürk Z.A., Karadağ G. (2014). Does Ramadan fasting alter body weight and blood lipids and fasting blood glucose in a healthy population? A meta-analysis. J. Relig. Health.

[19] Sadeghirad B., Motaghipisheh S., Kolahdooz F., Zahedi M.J., Haghdoost A.A. (2014). Islamic fasting and weight loss: A systematic review and meta-analysis. Public Health Nutr..

[20] Piaggi P., Vinales K.L., Basolo A., Santini F., Krakoff J. (2018). Energy expenditure in the etiology of human obesity: Spendthrift and thrifty metabolic phenotypes and energy-sensing mechanisms. J. Endocrinol. Investig..

[21] Gibney E.R. (2000). Energy expenditure in disease: Time to revisit?. Proc. Nutr. Soc..

[22] Heymsfield S.B., Harp J.B., Rowell P.N., Nguyen A.M., Pietrobelli A. (2006). How much may I eat? Calorie estimates based upon energy expenditure prediction equations. Obes. Rev..

[23] Poehlman E.T., Melby C.L., Goran M.I. (1991). The impact of exercise and diet restriction on daily energy expenditure. Sports Med..

[24] Lessan N., Saadane I., Alkaf B., Hambly C., Buckley A.J., Finer N., Speakman J.R., Barakat M.T. (2018). The effects of Ramadan fasting on activity and energy expenditure. Am. J. Clin. Nutr..

[25] McMurray R.G., Soares J., Caspersen C.J., McCurdy T. (2014). Examining variations of resting metabolic rate of adults: A public health perspective. Med. Sci. Sports Exerc..

[26] National Research Council (1989). Diet and Health: Implications for Reducing Chronic Disease Risk.

[27] De Jonge L., Bray G.A. (1997). The thermic effect of food and obesity: A critical review. Obes. Res..

[28] El Ati J., Beji C., Danguir J. (1995). Increased fat oxidation during Ramadan fasting in healthy women: An adaptative mechanism for body-weight maintenance. Am. J. Clin. Nutr..

[29] Alsubheen S.A., Ismail M., Baker A., Blair J., Adebayo A., Kelly L., Chandurkar V., Cheema S., Joanisse D.R., Basset F.A. (2017). The effects of diurnal Ramadan fasting on energy expenditure and substrate oxidation in healthy men. Br. J. Nutr..

[30] Lamri-Senhadji M.Y., El Kebir B., Belleville J., Bouchenak M. (2009). Assessment of dietary consumption and time-course of changes in serum lipids and lipoproteins before, during and after Ramadan in young Algerian adults. Singapore Med. J..

[31] Bakhotmah B.A. (2011). The puzzle of self-reported weight gain in a month of fasting (Ramadan) among a cohort of Saudi families in Jeddah, Western Saudi Arabia. Nutr. J..

[32] Al-Hourani H.M., Atoum M.F. (2007). Body composition, nutrient intake and physical activity patterns in young women during Ramadan. Singapore Med. J..

[33] Deranged Physiology. https://derangedphysiology.com/main/required-reading/endocrinology-metabolism-and-nutrition/Chapter%20318/physiological-adaptation-prolonged-starvation.

[34] Berg J.M., Tymoczko J.L., Stryer L. (2002). Food Intake and Starvation Induce Metabolic Changes. Biochemistry.

[35] Benedict F.G. (1915). Chemical and Physiological Studies of a Man Fasting Thirtyone Days. Proc. Natl. Acad. Sci. USA.

[36] Kerndt P.R., Naughton J.L., Driscoll C.E., Loxterkamp D. (1982). Fasting: The history, pathophysiology and complications. West. J. Med..

[37] Spriggs E.I. (1916). The Fasting Treatment of Diabetes. Br. Med. J..

[38] Cahill G.F. (1970). Starvation in man. N. Engl. J. Med..

[39] Danielsson E.J.D., Lejbman I., Akeson J. (2019). Fluid deficits during prolonged overnight fasting in young healthy adults. Acta Anaesthesiol. Scand..

[40] Korbonits M., Blaine D., Elia M., Powell-Tuck J. (2007). Metabolic and hormonal changes during the refeeding period of prolonged fasting. Eur. J. Endocrinol..

[41] Müller M.J., Enderle J., Pourhassan M., Braun W., Eggeling B., Lagerpusch M., Glüer C.C., Kehayias J.J., Kiosz D., Bosy-Westphal A. (2015). Metabolic adaptation to caloric restriction and subsequent refeeding: The Minnesota Starvation Experiment revisited. Am. J. Clin. Nutr..

[42] Al Junaibi A., Abdulle A., Sabri S., Hag-Ali M., Nagelkerke N. (2013). The prevalence and potential determinants of obesity among school children and adolescents in Abu Dhabi, United Arab Emirates. Int. J. Obes..

[43] National Health Services (NHS) https://www.nhs.uk/live-well/eat-well/the-eatwell-guide/.

[44] Office of Disease Prevention and Health Promotion https://health.gov/dietaryguidelines/2015/guideline/appendix2/.

[45] (2018). Balancing Energy in and Out; Nutrition Australia. http://www.nutritionaustralia.org/national/resource/balancing-energy-and-out.

[46] Hamdy O., Yuson B.N.M., Reda W.H., Slim I., Jamoussi H., Omar M. (2016). DaR Practical Guidelines; the Ramadan Nutrition Plan (RNP) for Patients with Diabetes.

[47] Vasan S., Thomas N., Bharani A.M., Abraham S., Job V., John B., Karol R., Kavitha M.L., Thomas K., Seshadri M.S. (2006). A double-blind, randomized, multicenter study evaluating the effects of pioglitazone in fasting Muslim subjects during Ramadan. J. Diabetes Dev. Ctries..

[48] Lessan N., Hannoun Z., Hasan H., Barakat M.T. (2015). Glucose excursions and glycaemic control during Ramadan fasting in diabetic patients: Insights from continuous glucose monitoring (CGM). Diabetes Metab..

[49] Hajek P., Myers K., Dhanji A.R., West O., McRobbie H. (2012). Weight change during and after Ramadan fasting. J. Public Health.

[50] Finch G.M., Day J.E., Welch D.A., Rogers P.J. (1998). Appetite changes under free-living conditions during Ramadan fasting. Appetite.

[51] Fernando H.A., Zibellini J., Harris R.A., Seimon R.V., Sainsbury A. (2019). Effect of Ramadan Fasting on Weight and Body Composition in Healthy Non-Athlete Adults: A Systematic Review and Meta-Analysis. Nutrients.

[52] Racinais S., Périard J.D., Li C.K., Grantham J. (2012). Activity patterns, body composition and muscle function during Ramadan in a Middle-East Muslim country. Int. J. Sports Med..

[53] Poh B.K., Zawiah H., Ismail M.N., Henry C.J.K. (1996). Changes in body weight, dietary intake and activity pattern of adolescents during Ramadan. Malays. J. Nutr..

[54] Lean M.E., Garthwaite P. (1995). Weight loss and longevity. Ann. Intern. Med..

[55] (2016). Ramadan Nutrition Plan. Diabetes and Ramadan International Alliance (DaR). https://www.daralliance.org/daralliance/en/dr/about-rnp.html.

[56] Shadman Z., Akhoundan M., Poorsoltan N., Khoshniat Nikoo M., Larijani B., Akhgar Zhand C., Soleymanzadeh M., Alsadat Seyed Rohani Z., Jamshidi Z. (2016). Nutritional Education Needs in Relation to Ramadan Fasting and Its Complications in Tehran, Iran. Iran. Red Crescent Med. J..

[57] Shadman Z., Poorsoltan N., Akhoundan M., Larijani B., Soleymanzadeh M., Akhgar Zhand C., Seyed Rohani Z.A., Khoshniat Nikoo M. (2014). Ramadan major dietary patterns. Iran. Red Crescent Med. J..

[58] Leiper J.B., Molla A.M., Molla A.M. (2003). Effects on health of fluid restriction during fasting in Ramadan. Eur. J. Clin. Nutr..

[59] Carroll H.A., Templeman I., Chen Y.C., Edinburgh R.M., Burch E.K., Jewitt J.T., Povey G., Robinson T.D., Dooley W.L., Jones R. (2019). Effect of acute hypohydration on glycemic regulation in healthy adults: A randomized crossover trial. J. Appl. Physiol..

[60] Turin T.C., Ahmed S., Shommu N.S., Afzal A.R., Al Mamun M., Qasqas M., Rumana N., Vaska M., Berka N. (2016). Ramadan fasting is not usually associated with the risk of cardiovascular events: A systematic review and meta-analysis. J. Family Community Med..

